# CORRELATES OF SOCIAL COHESION AMONG COMMUNITY-DWELLING OLDER PERSONS IN SOUTH-WESTERN NIGERIA

**DOI:** 10.1093/geroni/igac059.1111

**Published:** 2022-12-20

**Authors:** Eniola Cadmus, Lawrence Adebusoye, Eme Owoaje

**Affiliations:** College of Medicine/ University of Ibadan, Ibadan, Oyo, Nigeria; Chief Tony Anenih Geriatric Centre, University College Hospital, Ibadan, Oyo, Nigeria; University of Ibadan, Ibadan, Oyo, Nigeria

## Abstract

Existing research shows that older persons prefer to ‘Age in Place’ in their homes or community for as long as possible and are attached to both the physical space as well as the social environment. Despite growing attention to the relationship between social cohesion and health outcomes, few studies have focused on its correlates, especially in low- and middle-income countries, including Nigeria. This study identified the factors related to neighbourhood social cohesion among older adults aged >60years dwelling in a selected rural and urban community in Oyo State, southwestern Nigeria. Data were collected using a semi-structured, interviewer-administered questionnaire and analysed using Stata version 15. Overall, 1180 (588 rural and 592 urban) respondents were interviewed (mean age 74.2 ± 9.5 years). Over half (55.3%) of the respondents had an adequate level of social cohesion. In the rural environment, positive predictors of social cohesion were male gender [OR: 1.73 (95%CI: 1.10-2.73)], formal education [OR:2.70 (95%CI: 1.52-4.81)] being employed [OR:3.68 (95%CI: 2.50-5.42)], homeownership [OR: 1.82 (95%CI: 1.22-2.71)] and good self-rated health (SRH) [OR:2.73 (95%CI: 1.78-4.20)]. Among the urban respondents, factors predictive of good social cohesion were male sex [OR:1.68 (95%CI: 1.06-2.68)], being employed [OR:2.38 (95%CI: 1.67-3.41) and reporting good SRH [OR:2.13 (95%CI: 1.45-3.11)]. Older respondents were less likely to have good social cohesion compared with the younger respondents in the rural [OR:0.54 (95%CI: 0.35-0.84)] and urban setting [OR:0.68 (95%CI: 0.47-1.00)]. The study findings underscore the importance of socio-economic status and homeownership on older adults’ social cohesion and areas for targeted policy and intervention

